# Colorectal Cancer Immune Infiltrates: Significance in Patient Prognosis and Immunotherapeutic Efficacy

**DOI:** 10.3389/fimmu.2020.01052

**Published:** 2020-05-28

**Authors:** Liang Guo, Chuanlei Wang, Xiang Qiu, Xiaoyu Pu, Pengyu Chang

**Affiliations:** ^1^Department of Pathology, The First Hospital of Jilin University, Changchun, China; ^2^Department of Hepatobiliary Pancreatic Surgery, The First Hospital of Jilin University, Changchun, China; ^3^Department of Radiology, The First Hospital of Jilin University, Changchun, China; ^4^Department of Radiation Oncology and Therapy, The First Hospital of Jilin University, Changchun, China; ^5^Jilin Provincial Key Laboratory of Radiation Oncology and Therapy, The First Hospital of Jilin University, Changchun, China; ^6^Key Laboratory of Organ Regeneration and Transplantation of the Ministry of Education, Department of Radiation Oncology and Therapy, The First Hospital of Jilin University, Changchun, China

**Keywords:** colorectal cancer, immunotherapy, cancer immune milieu, lymphocyte, cancer prognosis

## Abstract

Colorectal cancer occurrence and progression involve multiple aspects of host immune deficiencies. In these events, immune cells vary their phenotypes and functions over time, thus enabling the immune microenvironment to be “tumor-inhibiting” as well as “tumor-promoting” as a whole. Because of the association of tumoricidal T cell infiltration with favorable survival in cancer patients, the Immunoscore system was established. Critically, the tumoral Immunoscore serves as an indicator of CRC patient prognosis independent of patient TNM stage and suggests that patients with high Immunoscores in their tumors have prolonged survival in general. Accordingly, stratifications according to tumoral Immunoscores provide new insights into CRC in terms of comparing disease severity, forecasting disease progression, and making treatment decisions. An important application of this system will be to shed light on candidate selection in immunotherapy for CRC, because the T cells responsible for determining the Immunoscore serve as responders to immune checkpoint inhibitors. However, the Immunoscore system merely provides a standard procedure for identifying the tumoral infiltration of cytotoxic and memory T cells, while information concerning the survival and function of these cells is still absent. Moreover, other infiltrates, such as dendritic cells, macrophages, and B cells, can still influence CRC prognosis, implying that those might also influence the therapeutic efficacy of immune checkpoint inhibitors. On these bases, this review is designed to introduce the Immunoscore system by presenting its clinical significance and application in CRC.

## Introduction

Colorectal cancer (CRC) is a cancer with a high incidence in industrialized countries. Epigenetic and genetic events are inherently involved in CRC pathogenesis (1). In addition, habits and customs also influence this process, such as a high-fat diet, excessive intake of red meat, smoking, and drinking. Currently, surgery, radiotherapy, and systematic therapy have become the standards of care for CRC patients (2, 3). With the introduction of these approaches, a multidisciplinary treatment decision can be made to manage CRC patients, and most patients can benefit from comprehensive therapies. As such, the 5-year survival rate of CRC patients has reached over 50% in most regions worldwide (4).

Traditionally, the TNM staging system is the most available tool for comparing disease severity and predicting the prognosis of CRC. As more advances and insights into CRC heterogeneity and molecular characteristics are gained, other indexes have been introduced to discriminate CRC prognosis, such as RAS or BRAF mutations, and the microsatellite status in tumors (5). More comprehensively, the molecular subtypes of CRC have been stratified by using next-generation sequencing (NGS) technology (6). Accordingly, the CRC patients in each subtype differ in their prognoses (6). Beyond a doubt, profiling of the CRC molecular characteristics will enable treatment decisions to be more precisely and personally made. However, currently, there is still a lack of valid evidence suggesting that NGS-based CRC diagnosis and treatment will improve the prognosis of patients.

Beyond identifying the molecular events occurring in tumor cells, more efforts have been made in profiling the tumor microenvironment in recent years. Herein, characterizing the immune status of tumors is more attractive, because cancer occurrence and progression exhibit a high association with deficiencies, such as immune defense, immune surveillance, and immune homeostasis. In the published studies, several methods concerning the identification of CRC immune status have been established, such as calculating the derived neutrophil to lymphocyte ratio (dNLR) (7), determining the Crohn's-like lymphoid reaction, peritumoral lymphocytic reaction, and intra-tumoral periglandular reaction plus the density of TILs (8), and evaluating the tumoral Immunoscore (9). Among them, the Immunoscore system is the most reliable because several lines of evidence have revealed that tumoral Immunoscores can independently determine CRC prognosis (9). Based on the ability of the T cell subsets, including Th1, cytotoxic T, and memory T cells, to cause tumor shrinkage, the densities of CD3^+^CD45RO^+^ memory T cells and CD3^+^CD8^+^ cytotoxic T cells either in the tumor center (CT) or in the tumor invasive margin (IM) were included into this system (9). Herein, if any region is strongly positive for memory T cells or cytotoxic T cells, senior pathologists will assign a score of 1, thus enabling Immunoscores to reach 0–4 points (9). In addition to predicting CRC prognosis, the system requires less techniques and costs; thus, clinicians and pathologists recommend it as a routine evaluation for CRC patients in the clinical setting (9).

Immunotherapy has opened a new era of cancer treatment. The therapeutic efficacies of immune checkpoint inhibitors targeting PD-1, PD-L1, and CTLA-4 are being investigated across cancers. Also being explored are the microsatellite instability (MSI) and deficient mismatch repair (dMMR) statuses, which appear to be credible biomarkers for selecting CRC patients who will benefit from immune checkpoint inhibition (10, 11). Critically, the T cells represented in the Immunoscore system have tumoricidal functions, and they serve as responders to immune checkpoint inhibitors due to their positive expression of PD-1 or CTLA-4. In this regard, can the Immunoscore become a biomarker for candidate selection in immunotherapy? Current data indicate that evaluating T and B cell densities in CRC tumors exhibits higher accuracy than evaluating PD-L1 expression in predicting the effectiveness of immunotherapy (12), because PD-L1 can be heterogeneously expressed within diverse regions of a tumor (12). Moreover, especially in metastatic CRC, the lesions can have variable Immunoscores (12). Meanwhile, other immune infiltrates, such as Tregs, dendritic cells, and macrophages, might also influence the immune landscape in a CRC tumor (13). This is merely a pitfall of the Immunoscore system. In this review, we will discuss all of the aforementioned issues.

## Immunoscore: An Independent Factor Determining CRC Prognosis

Tumoral infiltration of T cells has been to be a prognostic factor across several cancers (14). In the past two decades, studies have confirmed the association of high densities of CD3CT^+^IM^+^, CD45ROCT^+^IM^+^, GZMBCT^+^IM^+^ (granzyme B, a hallmark of cytotoxic T cells), and CD8CT^+^IM^+^ T cells with prolonged disease-free survival (DFS) and overall survival (OS) of patients with localized CRC (15–19) (Table 1). In addition, Immunoscores can be used to predict the prognosis of CRC patients with metastases. As with primary tumors, metastatic tumors with 3–4 points predict CRC patients with better prognosis than those tumors with 0–2 points (20). Alternatively, calculating primary and metastatic tumor Immunoscores jointly will be more precise in predicting CRC prognosis than calculating the score in one site (24). However, recent studies have confirmed that metastatic tumors commonly differ from each other, even in the same patient, suggesting that the metastatic tumor possessing the lowest score determines the DFS of the patient (12, 21).

**Table 1:** Landmark studies indicating the value of Immunoscore in predicting CRC prognosis.

**Author^**(REF)**^**	**Cancer** **location**	**TNM-stage**	**Sample** **size**	**Method**	**Main results**	**Multivariant analysis for the independence of Immunoscore**
Pagès et al. (15)	Colon cancer	I–III	2,681	IHC for CD3 plus CD8 either in CT and in IM	1. The risk of recurrence at 5 years: 8% (high score group) vs. 19% (intermediate score group) vs. 32% (low score group)	Immunoscore: A prognostic factor in prediction of DFS and OS independent of the parameters^A^
Mlecnik et al. (19)	CRC	I–III	599	IHC for CD45RO, CD8, CD3, and GZMB in tumor	1. Patients with low density of CD8^+^ T cells in their tumors have higher risk of relapse than those with high density of CD8^+^ T cells. 2. CD8^+^ T cells density in tumors inversely correlates with T-stage.	Immunoscore: A prognostic factor in prediction of DFS, DSS, and OS independent of the parameters^B^
Pagès et al. (18)	CRC	I–II	29	PCR for genes related to memory T, CD8 cytotoxic T, Th1 and Th2 orientation, inflammation, immunosuppression, and angiogenesis	1. Tumors with high densities of CD45RO^+^ cells show higher expressions of genes encoding CD8, GZMA, GZMK, perforin, T-bet, IFN-γ, IL12, and IL-18 than those with low density of CD45RO^+^ cells. 2. Tumors with high densities of CD45RO^+^ cells show lower expressions of genes associated with inflammation, Th2 orientation, and angiogenesis.	Both Immunoscore and bowel perforation are independent prognostic factor in prediction of DFS, DSS, and OS
			602	IHC for CD8 plus CD45RO either in CT or in IM	1. Patients with high densities of CD8^+^ and/or CD45RO^+^ cells in their tumors have significantly prolonged DFS and OS.	
Galon et al. (17)	CRC	I–IV	75	Microarray analysis for genes encoding T-bet, IRF-1, IFN-γ, CD3ε, CD8, granulysin, and GZMB	1. High expressions of genes encoding genes encoding T-bet, IRF-1, IFN-γ, CD3ε, CD8, granulysin, and GZMB inversely correlates with tumor recurrence.	Immunoscore: A prognostic factor in prediction of DFS and OS independent of the parameters^C^
			415	IHC for CD3, CD8 plus CD45RO either in CT or in IM	1. Patients with high densities of CD3^+^,CD8^+^ or CD45RO^+^ memory T cells in their tumors have significantly prolonged DFS and OS.	
Pagès et al. (16)	CRC	I–IV	75	PCR for mRNA encoding CD8, T-bet, IRF-1, IFN-γ, granulysin and GZMB	1. Tumors without VELIPI show higher levels of mRNA encoding mRNA encoding CD8, T-bet, IRF-1, IFN-γ, granulysin and GZMB than those with VELIPI.	Patients with high density of CD45RO^+^ cells in their tumors have improved DFS and OS than those with low density of CD45RO^+^ cells
			39	Flow-cytometry for CD8^+^CD45RO^+^ T cells in tumor	1. Tumors without VELIPI show higher amount of CD8^+^CD45RO^+^ T cells than those with VELIPI.	
			415	IHC for CD45RO in tumor	1. High density of CD45RO^+^ cells in tumor correlates with absence of VELIPI and early TNM-stage.	
Van den Eynde et al. (12)	mCRC	IV	603	IHC for CD3, CD8, CD45RO, FOXP3, CD20 and PD-L1 in tumor	1. Patients receiving preoperative systemic therapies present their metastases rather than primary tumors with higher densities of CD3^+^, CD8^+^, and CD45RO^+^ cells in IM than those without treatment. 2. Preoperative chemotherapy plus anti-EGFR is apt to increase the densities of CD8^+^ cell and PD-1^+^ cell in IM and CT of metastases, and FOXP3^+^ cell density in primary tumors. 3. Preoperative chemotherapy plus anti-VEGF therapies is apt to increase B cell density in primary tumors. 4. The metastases in a CRC patient differ in their Immunoscore. 5. The metastatic lesion bearing least amounts of immune infiltrates (CD3/CD8/CD20) has the highest risk of relapse in a mCRC patient. 6. Immunoscore is superior to PD-L1 in reflecting the immune infiltrates of metastases.	The DFS of a mCRC patient is highly associated with the metastases with least Immunoscore (CD3 plus CD8) or least T-B score (CD8 plus CD20)
Wang et al. (20)	CRCLM	IV	249	IHC for CD3 plus CD8 in IM and CT	1. CRCLM patients with high Immunoscore in their metastatic tumors have significant improvement in RFS and OS comparing to those with low Immunoscores after liver surgery.	Immunoscore: A prognostic factor in prediction of RFS and OS^$^ independent of the parameters^D^
Mlecnik et al. (21)	mCRC	IV	441	IHC for CD3, CD8, CD45RO, CD20 and FOXP3 in IM and CT	1. The metastatic lesion with the lowest Immunoscore (CD3 plus CD8) or T-B score (CD8 plus CD20) determines the DFS and OS of a mCRC patient. 2. Except for CD45RO and FoxP3, the densities of CD3^+^, CD8^+^ and CD20^+^ in IM and CT are significantly higher in TRG 1–3 tumors than in TRG 4–5 tumors after preoperative treatment.	Both Immunoscore and T-B score are prognostic factors in prediction of DFS and OS independent of the parameters^E^
Mlecnik et al. (22)	CRC	I–III	760	Integrative analysis for gene expression	1. MSI tumors commonly have higher expressions of genes encoding IFN-γ, IL-15, GNLY, CCL3, CCL16, and markers indicating cytotoxicity, CD8, Th1, Th2, and Tfh. 2. MSI tumors commonly have higher densities of cytotoxic T cell, B cell, and macrophage in IM and CT than MSS tumors. 3. MSI tumors commonly possess high frequency of frameshift mutations, immunoediting, and functional specific anti-tumoral T cells.	Immunoscore: A prognostic factor in prediction of DSS^**#**^, DFS^**#**^, and OS^**&**^ independent of the parameter^F^
			367	IHC for CD8 and CD45 RO in IM and CT	1. MSI tumors have high frequency of high Immunoscore than MSS tumors. 2. A subpopulation of MSS tumors can have high Immunoscore.	
Mlecnik et al. (23)	CRC	I–IV	314	Genomic profiling	1. M1 tumors show higher frequency of *VHL* and *FBXW7* deletions than M0 tumors. 2. M1 tumors significantly downregulate their expressions of genes participating in T cell activation, costimulation, proliferation, IFN-γ secretion, response to IFN-γ, type I interferon signaling pathway, antigen processing and presentation via MHC-I/II.	Either Immunoscore (CD3 plus CD8) or GZMB plus PDPN score discriminate OS of CRC patients with or without metastasis
			524	IHC for CD3, CD8, CD57, T-bet, CD45RO, CD68, CD1A, GZMB, and PDPN in IM and CT	1. M1 tumors commonly have lower PDPN^+^ lymphatic vessel density than M0 tumors. 2. M1 tumors commonly have lower densities of CD3^+^, CD8^+^, CD57^+^, T-bet^+^, CD45RO^+^, GZMB^+^, CD68^+^ than M0 tumors.	

A Patient age, sex, T-stage, N-stage, MSI/MSS, mucinous colloid type, VELIPI, poor differentiation.

B Patient sex, T-stage, N-stage, total number of lymph nodes, histologic grade, mucinous colloid type, occlusion, bowel perforation.

C T-stage, N-stage, histological grade/differentiation.

D Patient age, sex, primary tumor location, T-stage, interval from primary tumor resection to liver metastases, perioperative chemotherapy^$^, number of metastases^$^ (^$^showing independence in prediction of OS).

E Patient age, T-stage, N-stage, primary tumor location, preoperative treatment (Chemotherapy or plus anti-angiogenic therapy or anti-EGFR therapy), histological grade/differentiation, metastasis surgery R status (R0 or R1), number of metastases, synchronous, or metachronous metastasis, TRG, RAS status, and two-stage hepatectomy.

F Patient sex, T-stage, N-stage, histological grade, VELIPI^#^, Mucinous colloid type, tumor occlusion, tumor perforation and MSI status (^#^showing independence in prediction of DSS and DFS; ^&^showing independence in prediction of OS).

*CRCLM, colorectal cancer liver metastasis; CT, center region of tumor; mCRC, metastatic colorectal cancer; DFS, disease-free survival; DSS, disease-specific survival; RFS, relapse-free survival; OS, overall survival; IHC, immunohistochemical staining technology; IM, invasive margin of tumor; PCR, polymerase chain reaction technology; TRG, tumor remission grade; VELIPI, venous emboli and lymphatic and perineural invasion*.

The above data specify the basic role of the Immunoscore in predicting CRC prognosis. Here, the clinical significance of the Immunoscore in CRC prognosis determination is addressed. In order to illustrate this issue, we should take the MSI phenotype as a comparison, because this phenotype has been reported to be the immune subtype of CRC (6). In theory, CRC tumors with MSI phenotypes commonly possess high densities of tumoricidal T cells due to the abundance of neoantigens from frequent frame-shift mutations occurring in tumor cells (25). Moreover, prospective studies have confirmed the therapeutic effects of immune checkpoint inhibitors on metastatic CRC with the MSI phenotype (10, 11), and MSI testing has been recommended for selecting CRC patients who can benefit from immune checkpoint blockade (2). Concerning the prognostic value of MSI in CRC, a study reported that among all molecular subgroups of CRC, patients with MSI phenotypes can have their prognoses at a moderate level (6), however, their prognoses will get worst after tumor relapse (6). Moreover, when comparing the abilities of the Immunoscore and an MSI phenotype in predicting the prognosis of CRC patients, significant discrepancies still exist in predicting the prognosis of CRC patients; that is, the MSI phenotype suggests that patients with low scores (0–2 points) still exhibit shorter DFS and OS than those with high scores (3–4 points) (22). In this regard, Immunoscore exhibits the superiority to MSI phenotype in predicting CRC prognoses. In addition, patients with microsatellite stability (MSS) or an MSI phenotype also present comparable DFS and OS values when they share similar Immunoscores in their tumors, thus confirming that the prognosis of CRC patients depends on the Immunoscore rather than on MSI or MSS status (22). Actually, the Immunoscore exhibits value in predicting CRC prognosis regardless of several other factors, such as patient sex, tumor-associated occlusion or perforation, TNM stage, histologic grade, mucinous colloid type, vascular emboli of tumor cells, lymphatic invasion, perineural invasion, and the genomic alteration pattern of CRC cells (15, 22, 23). Thus, these data confirm that the Immunoscore is able to independently determine the prognosis of CRC patients.

## Immunoscore: Applications in the Clinical Setting

Evaluating tumoral Immunoscores indeed provides novel insights into the prediction of CRC prognosis. What are the latent applications of this system in clinical settings? The TNM staging system for comparing CRC severity mainly relies on indexes, including tumor invasion depth, number of involved regional lymph nodes, and the type of distant organs involved. However, it is common to observe that CRC patients differ in their prognoses even if they have the same disease stage. Referring to the relationship between Immunoscore and TNM stage, it has been revealed that the Immunoscore generally decreases as the TNM stage increases (26). However, Immunoscores are heterogenous even in CRC tumors of the same TNM stage (26). Moreover, albeit at a low incidence, a certain portion of cases at advanced stages still possess high Immunoscores in their tumors, favoring prolonged patient survival (26). In this regard, introducing Immunoscores into the TNM staging system should enable the prediction of CRC prognosis to be more informed. In particular, if patients at early stages have rapid disease progression, the Immunoscore will assist in this context. As documented, about 20–25% of CRC patients at early stages can have relapse in their disease after surgery, indicating the surgery alone is not sufficient for treating their disease (9). Herein, it has been reported that early-staged CRC patients with tumoral Immunoscore at 0–2 points exhibit high risks of disease relapse (18). Thus, adjuvant therapies are encouraged to be included to improve their prognosis (9). In fact, this situation is more applicable for patients at stage II (27), because the most heterogeneous Immunoscores are present at this stage (15). Nowadays, duplet chemotherapy has been used as an adjuvant therapy for high-risk stage II colon cancers, which are characterized by the presence of at least one of the criteria including pT4 tumor; G3; bowel obstruction or perforation; vascular, lymphatic, or perineural invasion on histologic specimens; and fewer than 12 nodes examined. Apart from these risk factors, we propose the Immunoscore should be taken into account to enable the treatment decision more precisely and personally, especially for those early cases without aforementioned risk factors but only with low Immunoscores in their tumors. Nevertheless, to determine the value of Immunoscores in combining adjuvant therapies or not in early-staged CRC, extensive work should be done in the future.

In addition to adding value to the TNM staging system, the tumoral Immunoscore can be boosted by using conventional treatment strategies. For example, neoadjuvant chemoradiotherapy (nCRT) is strongly recommended as a downstaging therapy for local advanced rectal cancer (LARC) (3). Herein, some retrospective studies have revealed that nCRT is able to increase the tumoral densities of CD8^+^ T cells among some LARC tumors (28–30). Tumor control after nCRT is mostly due to direct cytotoxic effects, T cell infiltration might be a by-product. Thus, it is reasonable to speculate that nCRT potentially improves the immune milieu of a CRC tumor because of the increased tumoral infiltration of CD8^+^ T cells. On this basis, a phase 2 trial was designed to investigate the downstaging efficacy of nCRT followed by immunotherapy in LARC, and the preliminary results report that ~30% of enrolled patients have achieved pathological complete remission (pCR) after receiving five cycles of nivolumab post-nCRT (31). Hopefully, such a strategy will cause significant clinical complete remissions (cCRs) among a portion of LARC patients. cCR certainly benefits those patients who are willing to undergo the “watch and wait” strategy rather than immediate surgery, because pooled analysis results reveal that the LARC patients with cCRs post-nCRT have a 5-year survival rate of 100% (32). Tumors in the rectum have a low incidence of MSI phenotype (33), and thus, monitoring the Immunoscore before and after nCRT will be helpful in the selection of patients who can potentially benefit from immune checkpoint inhibitors. At least, a study had revealed that CRC tumors with high Immunoscore have a significant overrepresentation of the frequency of cells expressing PD-1 in CT and IM, as well as increased expression of *PD-1* mRNA (22). More strikingly, this study also found that about 50% of MSS tumors could have a high Immunoscore (22). In this regard, Immunoscore can become an available biomarker in selecting the candidates benefiting from immune-checkpoint inhibitors.

## Immunoscore in Guiding Immunotherapy: Advantages and Pitfalls

Currently, the available biomarkers for immunotherapy success include PD-L1 expression by tumor cells, tumor mutational burden, and deficient mismatch repair (dMMR) and MSI phenotypes (34). In current clinical trials, CRC patients with dMMR or MSI phenotypes are mostly encouraged to receive immunotherapy. Yet, the data from phase 3 trials indicate that not all of these patients will acquire full benefit from immune-checkpoint inhibitors (10, 11), thus revealing a pitfall of using MSI or dMMR in the selection of immunotherapy candidates. Nevertheless, it has been proposed that the Immunoscore will provide perspectives in guiding the application of immunotherapy (9). Technically, similar to other biomarkers, the Immunoscore evaluation is easy to perform and involves immunohistochemistry staining (9). Moreover, retrospective data have confirmed that Immunoscores have higher accuracy than MSI status (22) and PD-L1 (12) in reflecting the immune status of CRC tumors. However, the Immunoscore system still exhibits drawbacks, because it contains no information concerning the survival, function, and metabolic processes of T cells or their interactions with surrounding substances in tumors (27). For example, IL-15 deficiency has been reported to impair the proliferation and survival of T cells in CRC tumors, potentially limiting an increase in Immunoscore (35). Currently, trials evaluating the accuracy of the Immunoscores in selecting immunotherapy candidates in CRC are lacking. Therefore, it is difficult to determine the shortcomings of this system in guiding the application of immunotherapy in CRC.

## Immune Infiltrate: Cueing the Immune Landscape of CRC

**Figure 1:**
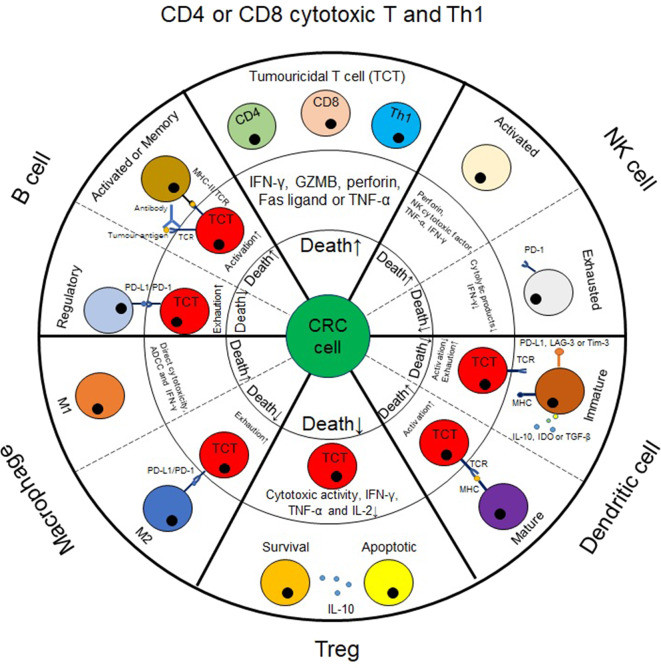
The impact of immune infiltrates on colorectal cancer cell death. In CRC tumors, immune infiltrates can impact CRC cell death, either directly or via tumoricidal T cells (TCT) and consequently affect tumor progression. For example, cytotoxic T cells, M1-like macrophages and NK cells can exert cytolytic effect on CRC cells. For other populations of cells, such as Treg, B cells, dendritic cells or M2-like macrophages, they generally impact CRC cell death by mediating the tumoricidal activity of TCT cells. Herein, Treg, regulatory B cells, immature dendritic cells and M2-like macrophages enable TCT cells to be exhausted, thus causing substantial progression in CRC tumors. By contrast, mature dendritic cells, activated or memory B cells generally induce TCT cell activation, thus causing tumor cell death.

In comparison with the Immunoscore, immune landscape profiling appears to be more promising, because it has been accepted that CRC-associated immune infiltrates can vary their phenotypes in a spatiotemporal manner (12, 13). Especially in metastatic cases, not only should the most prominent type of immune infiltrates be identified synchronously in primary and metastatic sites (12) but also the main biological processes at play in these cells should be targeted in a given period (36). For example, it has been demonstrated that in metastatic CRC, the tumor bearing the fewest tumoricidal immune infiltrates exhibits the highest risk of relapse (12). In this regard, it is reasonable to speculate that the responses to immunotherapy among metastatic tumors will vary. In the following sections, the potential impacts of several critical infiltrates on the effectiveness of immunotherapy and CRC prognosis will be discussed (Figure 1 and Table 2).

**Table 2:** Immune infiltrate-dedicated tumoral microenvironment and CRC immunotherapy.

**Infiltrate**	**TNM** **stage ↑**	**Immunopotent**	**Immunosuppression**	**Immunotherapy**	**Main effects**
Cytotoxic T cell	↓(9, 19)	√	NM	^**1**^**Pros in anti-PD-(L)1**	Cytotoxicity: Perforin, Fas ligand, TNF-α, GZMA/GZMB (37)
					Favorable prognosis: Cytolytic activity ↑ → Favorable prognosis (38)
					^**1**^**Critical responder to immune-checkpoint inhibitors**
Th1 cell	↓(9, 19)	√	NM	^**2**^**Pros in anti-PD-(L)1**	Tumoricidal function: IFN-γ-mediated type-1 immune response (39, 40)
					Favorable prognosis: Tumoral Th1 density and IFN-γ ↑ → Favorable provnosis (9, 19)
					^**2**^**Critical responder to immune-checkpoint inhibitors**
Treg cell	NM	√(Advantage)	√(Pitfall)	^**3**^**Pros in anti-CTLA4**	Advantage: Tumoral density of Treg ↑ Patient survival ↑(41–45)
					Pitfall: Tumoral density of Treg↑ → Poor tumor differentiation and more lymph node involvement (46)
					Apoptotic Treg cells are efficient in downregulating IFN-γ, TNF-α, and IL-2 by tumoricidal T cells (47).
					^**3**^**IL-10 induces CTLA-4 upregulation in Treg cells** (48)
B cell	NM	√(Advantage)	√(Pitfall)	^**4**^**Pros in anti-PD-(L)1**	Advantage: Tumoral densities of cytotoxic T and B cells ↑ → Patient survival ↑(12, 21)
					Pitfall: ^**4**^**PD-L1 by Breg cells elicits T cell exhaustion** (49)
					IL-35-producing B cells recruit MDSCs (50)
Natural killer cell	↓	√	NM	^**5**^**Pros in anti-PD-(L)1**	Tumoricidal function: Cytotoxicity and IFN-γ production (51)
					^**5**^**But NK cells are prone to exhaustion upon gut carcinogenesis with a phenotype of upregulation of PD-1** (51)
Dendritic cell	↓(52)	√(Advantage)	√(Pitfall)	^**6**^**Pros in anti-PD-(L)1**	Advantage: CD103^+^ myeloid DCs → CD4^+^ or CD8^+^ T cell activation (53)
					Pitfall: Plasmacytoid DCs → Treg cell induction (54)
					VEGF, PGE2, TGF-β, IL-10, IDO → DC maturation ↓, MHC-II and co-stimulatory molecules↓ → poor T cell activation (53–55)
					^**6**^**Immature DCs induce T cell exhaustion by PD-L1, Tim3, LAG3, IDO, IL-10 and TGF-β** (53–55)
Tumor-associated macrophage (TAM)	NM	√(Advantage)	√(Pitfall)	^**7**^**Pros in anti-PD-(L)1**	Advantage: Density of CD68^+^ M1-like TAMs in primary tumor↑ → Patient survival ↑(56–58)
					Pitfall: M2-like TAMs promote metastatic tumor progression by producing IL-35, IL-10, TGF-β, VEGF, and CCL2 (59–66)
					^**7**^**M2-like TAMs attract CD4**^**+**^ **or CD8**^**+**^ **T cells to cluster around them** (67), **thus eliciting T cell exhaustion by using PD-L1** (65)

*NM, not mentioned*.

### Cytotoxic T Cells

CD8^+^ T cells are the most potent cytolytic cell subset. Cytotoxic processes are carried out by several substances produced by CD8^+^ T cells, such as GZMB, perforin, Fas ligand (FasL), and TNF-α (37). Like CD8^+^ T cells, cytotoxic CD4^+^ T cells affect cell death via the Fas/FasL and GZMB/perforin pathways (37). In contrast to other CD4^+^ T subsets, cytotoxic CD4^+^ T cells have developmental programs of their own (68). In response to tumoral antigens, cytotoxic CD4^+^ T cells will increase in numbers (69). Moreover, a recent study confirmed that CRC patients with a favorable prognosis commonly have tumor immune cell infiltrates with increased cytolytic activities (38). However, the number of cytotoxic T cells decreases as TNM-stage increases in CRC (19).

In humans, CD8^+^ cytotoxic T cells (39), CD4^+^ cytotoxic T cells (69), and Th1 cells (69) are the most critical subsets producing IFN-γ. This cytokine functions by exclusively stimulating the JAK1/2-STAT1 pathway, which provokes several immunological processes, including macrophage activation, MHC-I/II pathway upregulation, costimulation, Treg cell inhibition, and Th1 cell differentiation and activation (39, 40). All these processes belong to the IFN-γ-mediated type-1 immune response, which profoundly elicits tumor remission. In parallel with cytotoxic T cells, a high density of tumoral Th1 cells predicts a favorable prognosis in CRC (70). Meanwhile, tumoral infiltrations of cytotoxic T cells and Th1 cells and IFN-γ upregulation serve as hallmarks indicating a good response to immune checkpoint inhibitors (71), because IFN-γ can upregulate PD-L1 and MHC-I expression by tumor cells (72). However, any deficiency leading to JAK1/2-STAT1 activation will distort the therapeutic efficacies of immune checkpoint inhibitors (73). This suggests that IFN-γ can synergize with the effectiveness of immune checkpoint inhibitors.

### Treg Cells

In humans, Treg cells are the most critical source of IL-10. This cytokine can exert multiple effects on immune cells, such as reducing the cytotoxic activity of CD8^+^ T cells, downregulating MHC-II-restricted antigens or CD80/CD86 expression by monocytes, inhibiting the synthesis of IFN-γ or TNF-α, and blocking the effector functions of dendritic cells and other CD4^+^ T cell subsets (Th1, Th2, or Th17 cells) (74, 75). In addition, IL-10 can upregulate the expression of CTLA-4 by Treg cells and strengthen their immunosuppressive potencies (48). However, results from several retrospective studies still support that tumoral infiltration of Treg cells potentially prolongs the survival of CRC patients (41–45). Experimentally, it has been confirmed that IL-10 is required for host immune surveillance and restricts carcinogenesis in the small intestine of mice (76). Strikingly, Treg cell densities in CRC specimens were found to inversely correlate with tumoral PD-L1 expression levels (77). In theory, reduced expression of PD-L1 will assist in protecting against T cell exhaustion. In fact, Treg cells are prone to apoptosis in CRC tumors (47). Functionally, apoptotic Treg cells are more efficient than live cells in downregulating the expression of IFN-γ, TNF-α, and IL-2 by tumoricidal T cells (47), while the pre-existence of apoptotic Treg cells in CRC tumors potentially distorts the therapeutic efficacies of immune checkpoint inhibitors (47). In this regard, apoptotic Treg cells impact more in the response of CRC to immune checkpoint inhibitors than living ones.

In contrast, other studies have found that tumoral Treg infiltration fails to predict the prognosis of CRC (46, 78). However, increased densities of Treg cells are associated with poor tumor differentiation and increased lymph node involvement (46). In fact, Treg cells contain heterogeneous subsets, and some of them contribute to CRC progression, such as CD8^+^ Treg cells (79), RORγt^+^ Treg cells (80) and IL-17-producing Treg cells (81). Typically, RORγt is pivotal in Th17 cell polarization (82). The expression of RORγt and IL-17 reflects the plasticity of Treg cells, especially in the presence of TGF-β, IL-1, IL-6, and IL-23 (58, 83, 84). As such, the density of Treg cells along with their associated cytokine profiles in tumors should be determined jointly, thus enabling an increase in the use of Treg cells in predicting CRC prognosis.

### B Cells

B cells include heterogeneous subsets and dominate antibody production, antigen presentation, and immunosuppression (85). In the healthy gut, B cells are widely distributed in the lamina propria and isolated lymphoid follicles (86). Like T cells, B cells require IL-15 to maintain their proliferation and survival (35). In the gut, they participate in epithelial barrier maintenance by producing secretory IgA (sIgA) while assisting in secretory IgM (sIgM) production by gut plasma cells (87). sIgA and sIgM are critical antibodies in protection against intestinal bacterial dysbiosis, which serves as an intrinsic factor in the induction of gut carcinogenesis (88).

When CRC occurs, the B cell subsets in peripheral blood, mesenteric lymph nodes and primary tumors differ in their phenotype indicating “activation” (89). Tumoral B cells form islet-like structures (90), which are induced predominantly by follicular helper T cells (10). In general, tumoral B cells are commonly activated and have memory phenotypes (91). They can activate tumoricidal T cells to manipulate cancer cell death due to their effectiveness in antigen presentation and co-stimulation. However, a recent study has revealed that a high density of tumoral B cells predicts favorable clinical outcome only in patients with right-sided colon cancer, rather than left-sided colon cancer or rectal cancer (90). Evaluating the densities of tumoricidal T cells, Treg cells, and B cells together might improve the accuracy of CRC prognosis prediction (12).

Not all B cells assist in the tumoricidal process. Upregulation of CXCL9 and CXCL10 in CRC tumors can attract regulatory B (Breg) cells as well, although such chemoattractants are also potent in recruiting tumoricidal T cells (49). Breg cells express PD-L1, thus severing as a route inducing T cell exhaustion (49). In addition, IL-35-producing B cells have been observed to increase their numbers upon CRC progression (50). This context will attract more myeloid-derived suppressive cells (MDSCs) (50).

### Natural Killer Cells

Natural killer (NK) cells also exert cytolytic function. At steady state, NK cells stand in the frontline against gut carcinogenesis (92). If CRC occurs, NK exhaustion will occur, resulting in a reduction in cytotoxic activity, IFN-γ downregulation, and PD-1 upregulation (92). Inherently, CRC cells can heterogeneously express NKG2D ligands, such as MICA and ULBP2/3, whereas MICB is always absent (93). As stress proteins, MICA, and MICB are crucial in mediating the activation of the recognition pathway in cytotoxic lymphocytes, whereas proteolytic shedding of these proteins leads to tumor evasion (94). Alternatively, if CRC cells are deficient in MHC-I expression or function, NK cells will limit their expansion and reduce the production of IFN-γ, GZMB, and perforin (51).

In parallel with cytotoxic T cells, NK cells have reduced numbers in tumors as TNM-stage increases (92). In metastatic CRC, it has been found that the number of tumoral NK cells is significantly less than that in peritumoral or normal tissue (95). On this basis, the role of tumoral NK cells in CRC prognosis has not been specified (95). However, it is at least known that NK cell infiltration into CRC tumors at advanced disease stages is difficult. To overcome this, quantitation of IFN-γ secretion by blood NK cells has been used to identify the cytotoxic status of NK cells, which provides a potential for screening patients at high risk of suffering CRC or monitoring disease progression (92). Alternatively, due to PD-1 upregulation in NK cells upon CRC occurring, immune checkpoint inhibitors should assist in preventing NK cell exhaustion.

### Dendritic Cells

Dendritic cells (DCs) are professional antigen-presenting cells in humans. In the steady-state setting, the hallmark functions of DCs include stimulating T or B cells, antigen presentation, and immunoregulation. Although the characteristics of human gut DCs are not well-understood (96), DCs located within the intestinal mucosa have been found to have the capacity to support homing of T and B cells from the periphery (96).

In humans, DC progenitors follow diverse paths to commit to plasmacytoid DC (pDC) and myeloid DC (also known as conventional DC, cDC) lineages (97). Their contributions on other immune cells are varied. For example, pDCs are prone to inducing Treg cell generation (98). Occasionally, pDCs can support the tumoricidal processes elicited by other immune cells, such as cDCs, T cells, B cells and NK cells (97). Among cDC subsets, cDC1 cells that are addicted to the transcriptional factor Batf3 for their polarization have been revealed to have the capacity to elicit CD8^+^ T cell-mediated immune responses via antigen cross-presentation (99). Further investigations support that CD103^+^ cDC1 cells are critical in processing tumor antigens to activate CD4^+^ or CD8^+^ T cells (100).

However, there is still no evidence indicating the impact of CD103^+^ cDC1 cells on CRC prognosis (41). The existing data indicate that tumoral infiltration of DCs is negatively associated with tumor stages, whereas the prognosis of CRC patients is diverse, because accumulations of DC cells with different phenotypes will result in poor DFS or OS (55). Inherently, immature or mature DCs can exert different effects on CRC progression (55). To help cancer cells escape from immune recognition and killing, DC differentiation and maturation can be inhibited by a panel of cytokines, including VEGF, prostaglandin-E2 (PGE2), TGF-β, IL-1β, IL-10, IL-13, and indoleamine-2,3-dioxygenase (IDO), which can originate from tumor cells or stromal cells (97, 98, 100). After contact with these cytokines, DCs downregulate MHC-II, and co-stimulatory molecules, thus resulting in poor T cell activation (97, 98). Moreover, immature DCs are proficient in inducing T cell exhaustion, because they can express PD-L1, Tim3, LAG3, IL-10, IDO, and TGF-β, thus strengthening immunosuppression in tumors (97, 98, 100).

### Tumor-Associated Macrophages

Tumor-associated macrophages (TAMs) are critical immune infiltrates in tumors. In general, they can be classified into two pools, namely, M1- and M2-like TAMs (54). In general, M1-like TAMs are inherently dedicated to antagonizing tumor progression, but M2-like TAMs are not [see details in Mantovani et al. (54)].

The phenotypes of TAMs are plastic. Although several studies have revealed that high densities of CD68^+^ macrophages in the tumoral IM predict favorable prognosis in patients with colon cancer (52, 53, 101), this is not the case in metastatic CRC. For example, more M2-like macrophages can be found in liver metastatic tumors than in primary sites (102). It has been revealed that by producing IL-35, liver TAMs can activate the STAT6-GATA3 axis of CRC cells to facilitate their colonization (102). In addition, exosomes from TP53-mutated CRC cells can induce the upregulation of IL-10, TGF-β, VEGF, and CCL2 by TAMs in a miRNA-1246-dependent manner (56). In addition to the immunosuppression elicited by VEGF, IL-10 (57) and TGF-β (58) are required for M2-like TAM polarization.

To a certain extent, M2-like TAM infiltration is associated with an increased incidence of CRC liver metastasis and promotion of disease progression in the liver. On the one hand, TGF-β-induced epithelial-mesenchymal transition in cancer stem cells serves as a critical route for CRC liver metastatic lesion formation (59). On the other hand, as a downstream molecule of TGF-β signaling, SMAD4 deficiency can lead CRC cells to upregulate the production of CCL15, which interacts with CCR1 on myeloid CD11b^+^MPO^+^ macrophages to recruit them into the liver (60). By producing metalloproteinase-9, CCR1^+^ macrophages assist in CRC invasion (60). Likewise, CCL2 attracts myeloid CD11b^+^Gr1^+^ macrophages to promote angiogenesis of metastatic tumors in the liver (61, 62). Therefore, retrospective studies have revealed that both CCL2 upregulation and CCR2^+^ TAM accumulation in tumors serve as factors indicating poor prognosis in patients with CRC liver metastasis (62, 63).

In parallel with CCL2 and CCL15, CCL5 serves as another chemokine that controls CRC progression (54). Functionally, CCL5 can interact with CCR5 on CRC cells to increase their proliferation, invasiveness, and metastasis (64). In this process, tumoral CD4^+^ and CD8^+^ T cells have been revealed to be the exclusive sources of CCL5 (64). In fact, TAMs are responsible for the events that CCL5 exploits, because they can produce CXCL9 and CXCL10 (64), which serve as critical attractants for T cell infiltration (64). CCL5 upregulation in metastatic tumors is accompanied by the accumulation of T cells, which cluster around TAMs (64). However, most of the T cells are of an exhausted phenotype (64). Functionally, TAMs can express PD-L1 (65). In addition, CRC liver metastatic lesions contain higher densities of PD-L1^+^ TAMs than primary sites (65). In this context, the concentrations of IFN-γ in CRC metastatic tumors are too low to enable the biological effects of this cytokine to be exerted (64). Therefore, the survival and tumoricidal functions of T cells can be impaired by TAMs. Additionally, CCL5-deficient mice bearing xenografted CRC display increased densities of tumoral CD8^+^ T cells (66), suggesting that CCL5 at least impacts T cell infiltration. In theory, T cell absence is believed to be a reason why tumor shrinkage is minimally induced by immune checkpoint inhibitors (103). If this is true, the CCL5-CCR5 axis could serve as a route that promotes CRC progression by excluding T cells. In fact, it has been proposed that CCL5-CCR5 blockade would potentially improve the anti-tumoral efficacies of immune checkpoint inhibitors (67). During this process, TAMs appear to be critical targets as well.

## Conclusion

The Immunoscore system provides new insights into reliably predicting CRC prognosis, especially as this system has potential for screening immunotherapy candidates. However, as several other tumoral infiltrates impact the efficacies of immune checkpoint inhibitors, much work is needed to determine whether the Immunoscore will become a superior biomarker indicating CRC immunotherapy. Alternatively, Immunoscore plus other diagnostic tools, such as MSI or dMMR appears to provide better CRC treatment, especially for immunotherapy.

## Author Contributions

PC, LG, and CW wrote this paper. XQ and XP prepared the tables of this review. PC conceived this topic of this review, and designed the logic flow.

## Conflict of Interest

The authors declare that the research was conducted in the absence of any commercial or financial relationships that could be construed as a potential conflict of interest.

## Abbreviations

CRC: colorectal cancer
NGS: next-generation sequencing
dNLR: derived neutrophil to lymphocyte ratio
TIL: tumor-infiltrating lymphocyte
IM: tumor invasive margin
CT: tumor center
MSI: microsatellite instability
dMMR: deficient mismatch repair
DFS: disease-free survival
OS: overall survival
MSS: microsatellite stability
nCRT: neoadjuvant chemoradiotherapy
LARC: local advanced rectal cancer
pCR: pathological complete remission
cCR: clinical complete remission
GZMB: granzyme B
FasL: fas ligand
sIgA: secretory IgA
sIgM: secretory IgM
Breg: regulatory B cells
MDSCs: myeloid-derived suppressive cells
NK: natural killer
DC: dendritic cell
pDC: plasmacytoid dendritic cell
cDC: conventional dendritic cell
PGE2: prostaglandin-E2
IDO: indoleamine-2,3-dioxygenase
TAM: tumor-associated macrophages
LPS: lipopolysaccharide
TLR4: toll-like receptor 4
CRCLM: colorectal cancer liver metastasis
CT: center region of tumor
mCRC: metastatic colorectal cancer
DSS: disease-specific survival
RFS: relapse-free survival
IHC: immunohistochemical staining technology
PCR: polymerase chain reaction technology
TRG: tumor remission grade
VELIPI: venous emboli and lymphatic and perineural invasion.
